# Correction: Progress in the discovery and development of anticancer agents from marine cyanobacteria

**DOI:** 10.1039/d6np90002j

**Published:** 2026-02-09

**Authors:** Hendrik Luesch, Emma K. Ellis, Qi-Yin Chen, Ranjala Ratnayake

**Affiliations:** a Department of Medicinal Chemistry, Center for Natural Products, Drug Discovery and Development (CNPD3), University of Florida 1345 Center Drive Gainesville Florida 32610 USA luesch@cop.ufl.edu; b Program in Cancer and Stem Cell Biology, Duke-NUS Medical School Singapore 169857 Singapore

## Abstract

Correction for ‘Progress in the discovery and development of anticancer agents from marine cyanobacteria’ by Hendrik Luesch *et al.*, *Nat. Prod. Rep.*, 2025, **42**, 208–256, https://doi.org/10.1039/D4NP00019F.

The authors regret that are errors in the structures shown in Fig. 2 and Fig. 4 of the published article. The changes made and the corrected figures can be found here.

In Fig. 2 the MMAF-based ADC is now depicted separately from MMAE-based ADCs because the linker is shorter compared with MMAE-based ADCs. The caption for Fig. 2 as included in the original article and here was correct at the time of publication. However, the authors would like to note that the MMAF-based ADC (Blenrep) has since been approved for clinical use.

In Fig. 4 the propyl carbon chain between dolastatin 15 and the maleimido group of compound **27a** has been updated to an ethyl carbon chain.

**Fig. 2:**
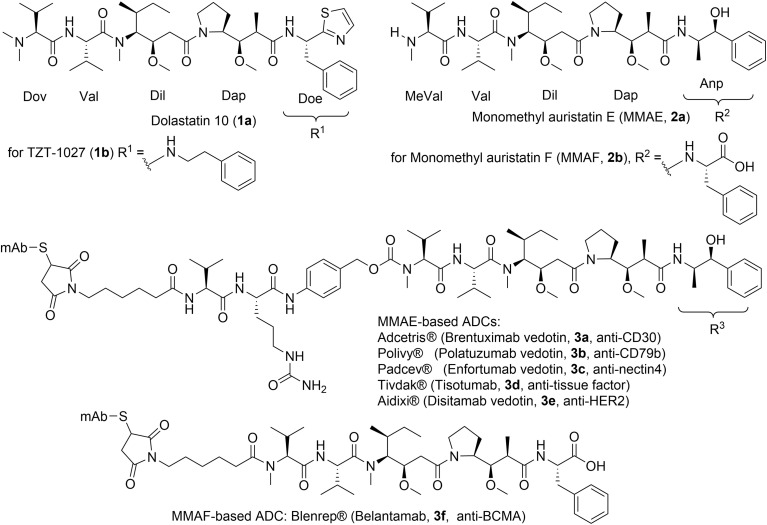
Structures of dolastatin 10 (**1a**), and the synthetic analogues of monomethyl auristatins developed as ADCs approved by FDA for clinical use. The MMAF-based ADC has been withdrawn.

**Fig. 4:**
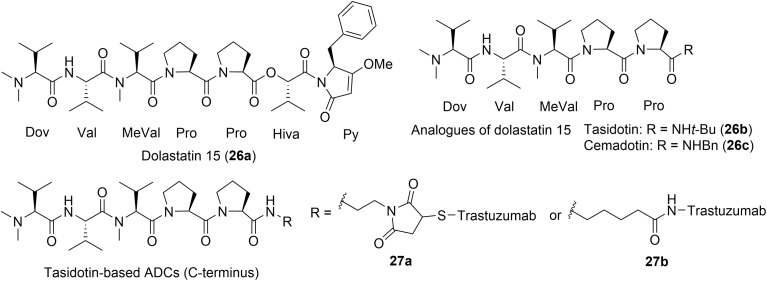
Dolastatin 15 (**26a**) and clinically evaluated analogues, including relevant ADCs that advanced to clinical trials.

The Royal Society of Chemistry apologises for these errors and any consequent inconvenience to authors and readers.

